# Assessing the Relationship Between Health Information Technology Use and Self-Rated Health Among Adults with Chronic Low Back Pain in the United States

**DOI:** 10.7759/cureus.39469

**Published:** 2023-05-25

**Authors:** Okelue E Okobi, Victor A Odoma, Okonkwo A Ogochukwu, Chika N Onyeaka, Cherechi G Sike, Rheiner N Mbaezue, Walter Iyare, Vaidehi Akhani, Chukwuma H Okeke, Soji Ojo, Adole A Moevi

**Affiliations:** 1 Family Medicine, Medficient Health Systems, Laurel, USA; 2 Family Medicine, Lakeside Medical Center, Belle Glade, USA; 3 Cardiology/Oncology, Indiana University (IU) Health Bloomington Hospital, Bloomington, USA; 4 Internal Medicine, College of Medicine, Ambrose Alli University, Ekpoma, NGA; 5 Public Health, Lagos State Ministry of Health, Lagos, NGA; 6 Family Medicine, Windsor University School of Medicine, Cayon, KNA; 7 Family Medicine, Abia State University Uturu (ABSU), Uturu, NGA; 8 General Medicine, Lugansk State Medical University, Luhansk, UKR; 9 Internal Medicine, Spartan Health Sciences University, Vieux Fort, LCA; 10 Informatics, Oracle Cerner Health, Houston, USA; 11 Psychiatry, University of Texas Health Science Center at Houston, Houston, USA; 12 Internal Medicine, Avalon University School of Medicine, Willemstad, CUW

**Keywords:** chronic low back pain, chronic low back pain (clbp), self-rated health, health information technology use, chronic, technology, health, information, pain, back

## Abstract

Objective: To assess the use of health information technology (HIT) among adults with chronic low back pain (CLBP) in the United States and to evaluate the relationship between HIT use and self-rated health.

Methods: The independent variable was the use of the internet to (1) fill prescriptions, (2) communicate with a healthcare provider, (3) look up health information, and (4) schedule a medical appointment. Respondents rated their health in the last 12 months as worse, about the same, or better. A Chi-square analysis was used to assess the use of HIT among those with CLBP; a logistic regression was used to determine predictors of HIT use; and an ordinal logistic regression was used to assess the relationship between HIT and self-rated health.

Results: As compared to those without CLBP, those with CLBP are more likely to use the internet to look up health information (58.9 vs. 53.8%, p-value<.001), refill prescriptions (13.9% vs. 10.5%, p-value<.001), and communicate with a healthcare provider (19.8% vs.15.3%, p-value<.001). Being employed and having a higher level of education were positive predictors of HIT use. As compared to other uses of the internet, using the internet to communicate with a healthcare provider was associated with higher odds of rating health as better compared to worse or about the same within the last 12 months.

Conclusion: Among adults with CLBP, a more affluent social status is associated with the use of HIT. Also, HIT is associated with a better health rating as compared to not using it at all. Further studies should assess the longitudinal relationship between HIT use and how adults with CLBP rate their health.

## Introduction

In 2018, the prevalence of chronic low back pain (CLBP) was 31% among United States (US) adults [1]. It is a major contributor to physical morbidity [2]. CLBP is more common in individuals who are older, obese, or have poor manual material handling skills [1,3,4]. Regardless of the approach to therapy, other factors are important in the care of patients with CLBP. Some of these factors include good patient-physician communication, access to healthcare, and high health literacy, all of which have been shown to affect the quality of care when dealing with patients with chronic medical conditions [5-8]. One tool that has garnered much interest in recent times in the care of patients with chronic medical conditions is health information technology (HIT), which may aid in addressing these factors.

In patients with CLBP, studies have reported positive results with the use of technology to improve posture and movements in the workplace [9]. However, few studies have examined how technology can assist patients with CLBP access to healthcare. Positive results have been reported in other populations with chronic medical conditions. Hou et al. reported that HIT use among individuals with diabetes mellitus was associated with an improvement in glycated hemoglobin levels, a well-known challenge in individuals with diabetes mellitus [10]. Kesiena et al. reported a positive association between HIT use and pneumococcal vaccine uptake among adults with heart disease [11], a group of individuals at risk of severe pneumococcal infections. Similarly, Young et al. reported a positive relationship between HIT use and adherence to inhalational corticosteroid use in asthmatics [12].

Patients with CLBP may benefit from HIT as it would help them establish a relationship with their healthcare providers, improve their knowledge about their health, and grant them easier access to healthcare. The objectives of this study are to evaluate the prevalence of HIT use among US adults with CLBP, assess the different ways they use HIT, and determine if it affects how they perceive their health.

## Materials and methods

Study design

This is a cross-sectional study utilizing the person- and adult-core data of the 2018 National Health Interview Survey (NHIS). The NHIS is a cross-sectional household interview survey of the non-institutionalized civilian population of the United States (US) conducted by the Centers for Disease Control and Prevention. Excluded from the survey are people in long-term care institutions, correctional facilities, and US nationals living in foreign countries. Sampling is a probability design, and interviewing occurs continuously throughout the year in a face-to-face format. The data contains sociodemographic and health information variables for US adults. Further details of this survey data are available elsewhere [13]. An IRB review was not required for this study because the data used for the analysis are publicly available.

Study population

We identified 25,397 adults who responded to a question about whether they had CLBP. Among those who responded to the question, 8062 (29.6%) reported having chronic low back pain.

Measures

Sociodemographic Variables

Age: 18 -39, 40 - 64, and ≥ 65 years; ethnicity: non-Hispanic White, Hispanic, non-Hispanic Black, non-Hispanic Asian, and other non-Hispanic race groups; gender: female and male; smoking status: current smokers, former smokers, and never smoked; body mass index: underweight, normal, overweight, and obese; employment status: employed and unemployed; and educational status: did not graduate high school, graduated high school, and graduated college.

Independent Variable: Health Information Technology

Respondents were asked if they use the internet to (1) fill prescriptions; (2) communicate with a health care provider; (3) look up health information; and (4) schedule a medical appointment. A composite variable was created from these, which was a cumulative use of the four HIT modalities. This variable had responses of at least one use of HIT, at least two uses of HIT, at least three uses of HIT, and at least four uses of HIT.

Dependent Variable: Self-Rated Health

Respondents were asked, “Compared with 12 months ago, would you say your health is better, worse, or about the same?”.This was represented as an ordinal variable in this study as worse, about the same, or better.

Statistical analysis

We analyzed 25,397 study participants who reported having or not having CLBP. Following NHIS protocols, sampling weights were applied during the data analysis to ensure the generalizability of the results. Descriptive statistics were used to analyze the sociodemographic characteristics of the study participants by CLBP status. The proportion of respondents who use HIT for four different purposes was determined using a Chi-square test, and multiple logistic regression was used to determine the sociodemographic predictors of HIT use by respondents with CLBP. Ordinal logistic regression was used to analyze the relationship between HIT and how respondents with CLBP perceived their health. The analysis was performed using STATA 14.0 with all p-values set at <.05.

## Results

Sociodemographic distribution of the study population

Table 1 below shows the sociodemographic characteristics of the study participants. Briefly, respondents with CLBP were mainly aged 65 years and older (36.8%), female (31.6%), non-Hispanic race groups (37.8%), had a BMI of 30 and above (36.9%), never smoked (41.7%), high school graduates (32.2%), and were unemployed (36.7%). Those without CLBP were mostly 18-39 years old (76.6%), male (72.0%), non-Hispanic Asian (80%), weighed 18-24.9 (75.3%), never used cigarettes (62.9%), had more than a bachelor’s degree (75.5%), and were employed (74.1%).

**Table 1:** Demographic characteristics of the study participants

Variable	No chronic back pain N (weighted frequency)	Chronic back pain N (weighted frequency)
Age (in years)		
18 – 39	5855 (76.6%)	1900 (23.4%)
40 – 64	6869 (67.8%)	3485 (32.3%)
65+	4611 (63.2%)	2677 (36.8%)
Gender		
Female	9248 (68.4%)	4609 (31.6%)
Male	8087 (72.0%)	3453 (28.0%)
Ethnicity		
Non-Hispanic White	12,000 (68.0%)	5842 (32.0%)
Hispanic	2297 (73.7%)	880 (26.3%)
Non-Hispanic Black	2051 (72.5%)	924 (27.5%)
Non-Hispanic Asian	1058 (80.0%)	268 (20.0%)
Other Non-Hispanic race groups	217 (62.2%)	148 (37.8%)
BMI		
<18.5	283 (74.8%)	117 (25.2%)
18.5 -24.9	5861 (75.3%)	2118 (24.7%)
25 -29.9	6029 (72.2%)	2511 (27.8%)
30+	5162 (63.1%)	3316 (36.9%)
Smoking status		
Does not smoke	3925 (62.9%)	2356 (37.1%)
Smokes	2129 (58.3%)	1626 (41.7%)
Level of education		
Graduated high school	11,000 (67.8%)	5794 (32.2%)
Bachelor’s degree	3986 (74.6%)	1418 (25.3%)
More than a bachelor’s degree	2428 (75.5%)	821 (24.5%)
Employment status		
Unemployed	6422 (63.3%)	4051 (36.7%)
Employed	11,000 (74.1%)	4008 (25.9%)

Prevalence of different uses of health information technology by chronic low back pain status (CLBP)

Table 2 shows the distribution of the study participants by the different uses of HIT. As compared to those without CLBP, those with CLBP use the internet more to look up health information (58.9 vs. 53.8%, p <.001), refill medical prescriptions (13.9% vs. 10.5%, p <.001), and communicate with a health care provider (19.8% vs. 15.3%, p <.001).

**Table 2:** Distribution of different uses of health information technology

Different uses of the internet	No chronic low back pain N (weighted %)	Chronic low back pain N (weighted %)	p
Use of the internet to look up health information	9147 (53.8%)	4524 (58.9%)	< .001>
Use of a computer to refill medical prescriptions	1828 ( 10.5%)	1062 (13.9%)	< .001>
Use of the internet to communicate with a healthcare provider	2686 (15.3%)	1488 (19.8%)	< .001>
Use of the internet to schedule a medical appointment	2684 (16.6%)	1276 (17.6%)	.116

Sociodemographic predictors of HIT use by those with chronic low back pain

Table 3 shows the sociodemographic predictors of HIT use by those with CLBP. Positive predictors of HIT use include being employed (odds ratio (OR)1.20 (1.04-1.39), p-value .015 and having a higher educational level (high school graduate, OR 3.45 (2.90-4.10), p-value <.001 and college graduate, OR 10.98 (9.87-13.58), p-value <.001). Negative predictors of HIT use include older age (40-64 years, OR 0.67 (0.57-0.80), p-value <.001 and 65 years +, OR 0.25 (0.21-0.31), p-value <.001), male sex OR 0.56 (0.49-0.64), p-value <.001, and race (black OR 0.73 (0.59-0.80), p-value .003 and Asian OR 0.74 (0.55-0.8036), p-value .036).

**Table 3:** Predictors of health information technology use in any form by respondents with chronic low AOR: adjusted odds ratio; CI: confidence intervals

Sociodemographic predictors	AOR 95% CI	p-value
Age		
< 40 years (reference)	1.00	
40 – 64 years	0.68 (0.58 – 0.81)	< .001>
65 years +	0.28 (0.23 – 0.33)	< .001>
Gender		
Female (reference)	1.00	
Male	0.56 (0.49 – 0.64)	< .001>
Race		
White (reference)	1.00	
Black	0.72 (0.59 – 0.88)	.002
Asian	0.74 (0.55 – 0.98)	.035
Others	0.79 (0.60 – 1.04)	.095
BMI, kg/m^2^		
<18.5 (reference)	1.00	.198
18.5 -24.9	1.44 (0.83 – 2.50)	.065
25 -29.9	1.68 (0.97 – 2.91)	.065
30+	1.75 (1.00 – 3.05)	.050
Level of education		
Did not graduate high school (reference)	1.00	
Graduated high school	3.49 (2.94– 4.14)	< .001>
Graduated college	11.33 (9.17– 14.00)	< .001>
Employment status		
Unemployed (reference)	1.00	
Employed	1.22 (1.05 -1.41)	.008

Self-rated health of adults with chronic low back pain (CLBP) who use health information technology

The crude and adjusted odds ratios of how adults with CLBP who use health information technology rate their health are shown in Table 4 below. The odds of rating their health as better than bad or about the same if they use the internet to communicate with a healthcare provider compared to not using the internet to communicate with their healthcare provider were 1.62 (1.35-1.94), p-value <.001. This odds ratio was reduced after adjusting for covariates to 1.55 (1.28-1.88), p-value <.001. The odds of rating their health as better than bad or about the same if they use the internet to look up health information compared to not using the internet to look up health information were 1.47 (1.26-1.72), p-value <.001. This odds ratio was reduced after adjusting for covariates to 1.31 (1.11-1.56), a p-value of .002. The odds of rating their health as better rather than bad or about the same if they use the internet to schedule a medical appointment were 1.34 (1.11-1.61), p-value .002. This odds ratio also reduced after adjusting for covariates 1.23 (1.02-1.49), p-value .034. There was no significant association between using the internet to refill medical prescriptions and how respondents with CLBP rated their health.

**Table 4:** The association between self-rated health and different uses of the internet among adults with chronic low back pain a) Adjusted for age, gender, education, race, and employment; b) Adjusted for age, race, education, employment, and BMI; c) Adjusted for age, gender, education, and employment

Different uses of the internet among adults with chronic	OR 95% CI	p	AOR 95% CI	p
Use of the internet to look up health information	1.47 (1.26 - 1.72)	< .001>	1.31 (1.11 - 1.56)^a^	.002
Use of a computer to refill medical prescriptions	1.14 (0.91 – 1.42)	.253	1.11 (0.89 – 1.39)^b^	.369
Use of the internet to communicate with a healthcare provider	1.62 (1.35 – 1.94)	< .001>	1.55 (1.28 – 1.88)^c^	< .001>
Use of the internet to schedule a medical appointment	1.34 (1.11– 1.61)	.002	1.23 (1.02 – 1.49)^c^	.034

## Discussion

The purpose of this study was to evaluate the prevalence of HIT use among US adults with CLBP given the documented benefits in other populations, assess the different ways they use HIT, and determine if it affects how they perceive their health. The main findings of this cross-sectional study were that 62.7% of those with CLBP use HIT, and they do so mostly to look up health information. In addition, being employed and having an increasing educational level positively predicted the use of HIT in any form, while being older than 39 years of age, having male sex, and being Black or Asian negatively predicted the use of HIT in any form. Overall, using the internet to communicate with a health care provider was associated with higher odds of rating health as better as compared to worse or about the same as compared to other uses of the internet.

This study showed that two in three individuals with CLBP use HIT in any of the four forms evaluated in this study, which is relatively higher when compared to the prevalence of HIT use in other studies. This is most probably due to the composite HIT term used during the analysis, as most studies about HIT use tend to evaluate one form of HIT use. We did, however, find out that with regards to the individual forms of HIT, 58.9% of the respondents who reported having CLBP used the internet to look up health information as compared to the other uses of HIT. This prevalence is comparable to that reported for the use of the internet to look up health information in two different studies. In the first study, 51.3% of the participants with heart disease used the internet to look up health information, compared to 58.8% in this study, while other uses of HIT had a lower prevalence [11]. In the second study, 53.7% of participants with skin cancer reported using the internet to look up health information [14]. A higher prevalence of the use of the internet to look up health information was, however, reported by Koo et al. among Taiwanese nationals (64.4%) [15].

In contrasting reports, another study reported a lower prevalence of the use of the internet for health information. In that study, 32.9% of Vietnamese adults reported using health information technology to obtain information online [16]. Although we found a high prevalence among US adults with CLBP, efforts to increase usage are needed given the reported benefits. In other populations, the use of the internet to look up health information was associated with an increased likelihood of paying a visit to their doctors [16]. The use of the internet to look up health information has also been associated with an increased likelihood of conformance with best clinical practices [17]. Despite this comparable prevalence, more effort is needed to increase the use of HIT among individuals with chronic low back pain due to HIT’s potential to ease access to health care.

A higher level of education and being employed, both of which are indicators of a higher socioeconomic status, were positive predictors of HIT usage among US adults with CLBP. Our findings were consistent with those of other studies. Li et al. reported that a higher level of education, female sex, and a younger age were associated with higher odds of seeking information online [18]. Invariably, other studies reported that a lower level of education was associated with a lower likelihood of HIT use in the form of looking up health information [19,20]. These findings may be related to the effect of health literacy on the use of technological tools. A higher level of health literacy, which is related to employment, and a higher level of education has been found to confer a higher likelihood of HIT use [21-23]. In a 2017 study of Korean adults by Sam Oh et al., they reported that for every one-unit increase in health literacy, the odds of seeking health information increased by 1.18 [24].

Racial minorities, male sex, and older age negatively predicted the use of HIT in any form. Similarly, these sociodemographic factors have been reported in other studies as associated with a lesser likelihood of HIT [19,25]. However, contrasting findings were reported in a study involving osteopathic medical students. Jacobs et al. reported that male sex was associated with a readiness to use HIT, with other factors such as prior exposure to technology and favorable attitudes towards HIT use also predicting HIT use [26], factors not considered in this study. This study, however, had a smaller sample size (n = 604) as compared to our study (n = 25, 397). These findings may mean that affluent individuals who do not belong to a racial minority group and are more health literate may likely benefit from health interventions related to health information technology. This is due to the increased odds of them being more likely to use these interventions, which may worsen the existing health disparity [27].

Interestingly, HIT usage was associated with positive self-rated health among individuals with CLBP. Among other populations, a better self-health rating has also been associated with the use of the internet, as reported in a study on the older Norwegian population [27]. Similar findings were reported in a cross-sectional study of older Swedish adults, and in that study, it was suggested that by using the internet, people can be better informed and educated [28]. We found that as compared to other uses of HIT, those who use the internet to communicate with their healthcare provider had the highest odds of rating their health as better as compared to worse or about the same. An explanation for this may be a result of the empowerment people feel when they have good communication with their healthcare providers [29]. Perhaps measures that encourage individuals who have CLBP to use HIT may lead to better interaction with the healthcare system and help lessen the burden of this mortality.

Strengths and limitations

This study used data from the NHIS, which is representative of the United States population. It used sampling weights during the analysis, which allows for the generalization of the results and eliminates non-response bias. This study evaluated an interventional tool that may become important in the management of CLBP given the rising burden and increased use of this tool in other areas. Despite the mentioned strengths, some limitations are worthy of mention. This was a cross-sectional study, so causation cannot be implied. In addition, this study used self-reported responses for questions about CLBP, which entertains a recall bias. The implication of this is that not all those who reported having CLBP may have low back pain for that long, as could also be said of those without CLBP. The NHIS data did not contain a more specific use of the internet. For instance, those who use the internet to access health information may be interested in health information about other health conditions they may have, which may not necessarily be about back pain. Likewise, those who use the internet to refill medical prescriptions may have requested other non-CLBP medications and the same non-specificity should be noted about the other uses of HIT studied.

## Conclusions

In conclusion, two in three US adults with CLBP use health information technology, and they use it mostly to look up health information. Higher socioeconomic status is associated with HIT use in people with CLBP, while male sex, increasing age, and being a racial minority were negatively associated with HIT use. This is similar to reported predictors of HIT usage in other populations. More effort is needed to address financial and racial disparities in access to health information technology. These efforts may, in turn, be beneficial in improving access to health care in general. Further studies should evaluate the specific uses of health information as it pertains to CLBP.
